# Allied health workforce development for participant-led services: structures for student placements in the National Disability Insurance Scheme

**DOI:** 10.1186/s12909-023-04065-y

**Published:** 2023-02-06

**Authors:** Stacie Attrill, Kristen Foley, Hailay Abrha Gesesew, Chris Brebner

**Affiliations:** 1 School of Allied Health Science and Practice, University of Adelaide, Adelaide, Australia; 2 College of Nursing and Health Sciences, Flinders University, Adelaide, Australia; 3 College of Medicine and Public Health, Flinders University, Adelaide, Australia; 4 Research Centre for Public Health, Equity, and Human Flourishing, Torrens University Australia, Adelaide, Australia; 5 School of Public Health, College of Health Sciences, Mekelle University, Mekelle, Tigray Ethiopia

**Keywords:** Clinical education, Health workforce, Allied health, Individualised funding, Work-integrated learning, Giddens’ structuration theory

## Abstract

**Background:**

Health, disability, and community services are increasingly transitioning from government-led to participant-led funding models, which intend to increase choice and control for service users. Allied health practitioners, who provide many frontline services within the resultant marketised environment, must adjust their knowledge and skills to meet participants’ expectations. However, future workforce strategies to address allied health student capabilities to provide these services have received limited attention. This study explored shifting understandings and practices related to allied health student placements during the implementation of a participant-led funding model within the Australian disability sector: the National Disability Insurance Scheme (NDIS).

**Methods:**

Data for this study came from a two-year disability workforce project exploring allied health placements. Service providers, participants, university representatives, disability advocates and students participated in 48 interviews and two focus groups to provide perspectives on allied health workforce and student placements. The findings result from secondary deductive analysis undertaken following project completion that used Gidden’s (1984) Structuration Theory as a conceptual lens to identify structures and actions related to the marketised service environment that influenced how allied health student placements were undertaken.

**Results:**

The findings were organised using two Structuration concepts: knowledgeability, and duality of structure. These described how service providers, supervisors and students understood, legitimised and prioritised placement activities, and how these structures influenced and were influenced by the actions of stakeholders across NDIS settings, contexts and time. Initially, existing placement structures were not compatible with new structures emerging in the disrupted NDIS service environment. However, over time, and responding to new knowledgeability of service providers, supervisors and students, placement structures were identified, monitored and adjusted to reflect perspectives of all stakeholders.

**Conclusions:**

Participant-led funding invoked structural changes in disability service provision that transformed how stakeholders understood placements and the role of students in service provision. Whilst there were new opportunities for placement, tensions were identified in how learning activities can be enacted within a marketised system in which resources are aligned to participant needs, and structures for workforce development and learning activities are less visible. Further conceptualisation of how student learning and workforce development activities can fit with contemporary funding models is necessary to meet participant, service provider and student needs.

**Supplementary Information:**

The online version contains supplementary material available at 10.1186/s12909-023-04065-y.

## Background

In several Western countries, government funding for health, disability and community services is transitioning from government-led, to participant-led funding models that intend to provide greater choice and control to participants through marketising and diversifying practice and services [1–3]. Allied health practitioners provide many services that participants seek via their funding, but limited attention has been paid to workforce development strategies that support practitioners to develop the knowledge, skills and capabilities for these changed environments [4]. Workforce development in allied health includes student placements that are critical opportunities for students to develop and apply relevant knowledge and skills under supervision, in practice with clients in contemporary service settings [5]. However, student placement activities may not readily translate into new service provision models or specific practices developed in response to participant-led funding [6]. This study explores how allied health students and disability service providers view student placements operating within a newly marketised participant-led funding environment as a workforce development strategy.

Participant-led funding models respond to the rights of individuals to make autonomous decisions about their preferences for services and care [2, 3]. As participants enact choice and control, the consequential marketisation both disrupts and influences service models and outcomes as these respond to consumer preferences and buying behaviours [1, 7, 8]. New services that participants prefer are introduced to the market, whilst services that do not meet participant needs are removed or adjusted [1, 9]. This marketised environment in turn, requires practitioners to develop knowledge, skills and capabilities that align with participants’ preferences [10, 11]. In particular, allied health practitioners, who provide frontline diagnostic, relational and therapeutic services, require new capabilities related to emerging services that differ to those offered with traditional government-led funding [5].

Strategies to develop a sufficient, prepared and competent workforce are needed to meet participants’ emerging needs [10–12]. However, typical health workforce planning and development strategies are derived from population-level methods that give limited attention to the skills and capabilities needed for emerging service provision and practice [12]. Further, for students who comprise the future allied health workforce, practice skills and competency development are directed by universities and broader professional accreditation needs [13], and intentional workforce development strategies that attend to the potential for participants to enact choice and control are not consistently included in learning activities [5, 10, 11].

In allied health, practice knowledge and skills are typically developed in pre-qualification placements situated within service settings [14, 15] to expose students to practice with service users and provide them opportunities to engage with organisational cultures, systems and processes. These placement experiences are critical for workforce development as they shape students’ professional socialisation, future career and employment aspirations [14, 16, 17]. Service providers often utilise placements as a strategy to engage and recruit future allied health staff and influence their workforce [5, 15]. Allied health students are typically supervised by allied health practitioners who work for the service provider hosting the placement [15, 18]. Supervision requires practitioners to engage in education activities that facilitate student learning, but are not always directly related to service provision [19, 20]. This includes observing and providing feedback to students, modelling and facilitating professional practices and clinical decision making [19, 21], enacted through a triadic relationship, where students are provided opportunities to work with clients, mediated by the supervising practitioner [22]. For students and supervisors, placement learning activities and processes take time and require a high cognitive load [21], however, this time is not explicitly included in participant-led funding, as the activities do not directly relate to participant services [1, 8]. Thus, services operating within marketised environments must identify strategies to facilitate student placements amidst funded participant activities to reduce financial impost. Research has not yet explored how workforce development can be facilitated for participant-led service models, nor how student placements, as part of workforce development, can be enacted within a marketised service environment.

Using the transition to a participant-led funding model for disability services in Australia, the National Disability Insurance Scheme (NDIS) as a case study, this paper uses Giddens’ 1984 Structuration theory [23] to unpack meanings, practices and positions related to allied health student learning in placements and broader impacts for workforce development. In relation to service provision, Giddens outlined dynamic interactions between *individuals in the system* (i.e. practitioners, NDIS participants, students and other stakeholders); and *structures in the system*, including the meanings, rules, processes and resources that guide an individual’s actions. Over time, as interactions are reproduced, individuals use their emerging knowledge and skills to interpret these as new structures which become progressively internalised and routinised. These structures then guide individuals’ everyday and future choices, helping them predict what will occur in the system in response to their actions. For example in a NDIS service, the respective knowledgeability of students, practitioner supervisors and NDIS participants about allied health placements produce structures that guide: a student’s actions to develop and practice the skills they understand as important; the supervisor’s actions to facilitate this learning whilst ensuring quality services for participants and adhering to organisational processes; and the actions of participants in contributing to student learning activities as part of their service.

Giddens theorised a co-productive relationship between individuals and structures that also facilitates them to renew and reproduce social systems and practices through their actions. Individuals can modify structures by reflecting and purposefully acting outside their usual rules or meanings. These may then become new structures if other stakeholders in the system reproduce and routinize them. For newly marketised services, this ‘structuration’ enables us to explore *why and how* practices are produced, reproduced, or not produced, by exploring how individuals interface with structure through the service environment. This is useful to explore emerging and continuing allied health student education practices situated with disability service providers who were, themselves transitioning to participant-led funding structures.

Therefore, this paper uses Structuration Theory [23] to conceptualise the research questions:

1. What shifts in structures and practices did service stakeholders involved with allied health student placements identify during the NDIS transition? and,
2. What do service managers and practitioners perceive as the enablers and barriers to quality practice placements for allied health students in a participant-led funding context?

## Methods

### Study design

Data for this study were derived from a two-year participatory action research project [24] that designed, piloted and evaluated allied health placement models for NDIS service providers (see [5] for a detailed methodology). The project was conducted in South Australia during a period of transition from government-led funding for disability service providers to a participant-led NDIS funding model. As the NDIS implementation had reduced the availability of allied health placements in disability services, this project was funded by the South Australian government to facilitate NDIS workforce development through identifying placement opportunities.

The project was overseen by a Project Advisory Group (PAG), comprised of 15 members representing disability services, government agencies, universities, allied health practitioners and the research team. Members were recruited through a purposive, network-based approach to provide diverse input regarding allied health workforce development in the newly marketised disability context. The PAG met on four occasions during the project to provide governance, endorse actions, offer iterative feedback and verify the project findings.

### Participants

In the first phase of the project, the research team purposively identified stakeholder organisations and other selected individuals from prior university and research team networks to provide insight about NDIS service provision, participation, student supervision, and the experience of being an allied health student on placement. Initially, representatives of each organisation or selected individuals were emailed by a member of the research team who had no prior relationship with them. Organisational representatives then provided consent for their employees to participate and provided study information to their employees. Individuals then emailed their written consent to a research team member who had not provided the initial information. These processes ensured that any coercive influence from prior relationships was minimised, and that employers were not aware of individuals who participated. Further relevant participants were invited as they were identified through the action research process. In total, 49 participants consented for the project, and 38 of these participated in one or more interviews. Some participants participated in multiple research activities (i.e. interview/s and PAG). The role, number of participants, occasions and range of interview method are represented in Table 1.

**Table 1:** Stakeholder role and number of participants

Stakeholder role	Identifier for Findings	Number of participants	Interview method	Number of interviews
Service providers: allied health practitioners	Service Provider	14	Semi-structured interview	22
Service providers: Chief Financial Officers	Admin	3	Semi-structured interview	4
University allied health placement staff	UniAHP	5	Semi-structured interview	7
Disability consumer group advocates	Adv	2	Semi-structured interview	4
NDIS participants	Participant	2	Semi-structured interview	2
Allied health students	Student	12	Semi-structured interview	7
			Focus group interview	2
Total		38		48

Allied health service providers, university representatives and students were occupational therapists, physiotherapists, and speech-language pathologists. These professions were identified during project planning as principal allied health services in the NDIS. University staff were allied health academics and practitioners who co-designed placement models with service providers to meet consumer and service needs. Chief Financial Officers were administrators who managed service provider business and financial functions, who were critical to ensure that placement models were sustainable. Disability consumer group advocates and NDIS participants provided information about impact of the NDIS implementation on consumers, service providers and the sector.

### Data gathering

Cycles of data were gathered over four iterations of interviews conducted over 2 years [5, 24]. The project team developed initial interview guides for each stakeholder group informed by a rapid literature and policy review addressing the NDIS funding and service provision models, allied health education and workplace learning (see supplemental files 1–7). The research team co-constructed the interview guides, and adjusted them iteratively during project meetings to respond to new action cycles, and in accordance with themes identified from concurrent data analysis. Interviews discussed the nature of the allied health workforce required for NDIS services and workforce gaps; how the workforce could enable NDIS participants to achieve their goals; opportunities for student placement activities within NDIS services; students’ experiences completing placement within NDIS settings; and perspectives on NDIS business and service models. Different schedules were used for different stages of the project and stakeholder groups.

Data were gathered through 46 individual semi-structured interviews and two student focus group interviews (see Table 1). Interviews ranged from 25 to 45 minutes, at times and locations convenient to the participants, and were conducted by five members of the research team trained in qualitative interviewing. There were 32 in-person interviews, including the two student focus group interviews, and 16 phone interviews. Ten participants were interviewed twice across different action cycles, with an intervening period varying from 6 to 18 months. For students, focus group and semi-structured interviews were used to optimise the number and availability of students who participated. Interviews were audio-recorded, transcribed verbatim, and the de-identified data were managed using NVivo versions 11 and 12 [25].

### Data analysis

The data reflected the iterative knowledge gathered through interviews across the action research cycles. To explore the structures and practices germane to marketised service provision contexts, the data were analysed as a single whole dataset, rather than being segmented according to the iteration or chronology of data gathering. The thematic framework method [26–28] utilised to guide analysis occurred in two phases:

1. An initial inductive thematic analysis conducted iteratively during the project, informed subsequent action cycles to inform the broader workforce project. Details of the methods, interpretation and analysis of this inductive analysis are published elsewhere [5].
2. A sequential deductive analysis using concepts from structuration theory [23] to identify structures and actions related to the newly marketised service environment, and how these influenced allied health placements and workforce development. Data from this deductive analysis are reported and interpreted in this paper.

**Fig. 1:**
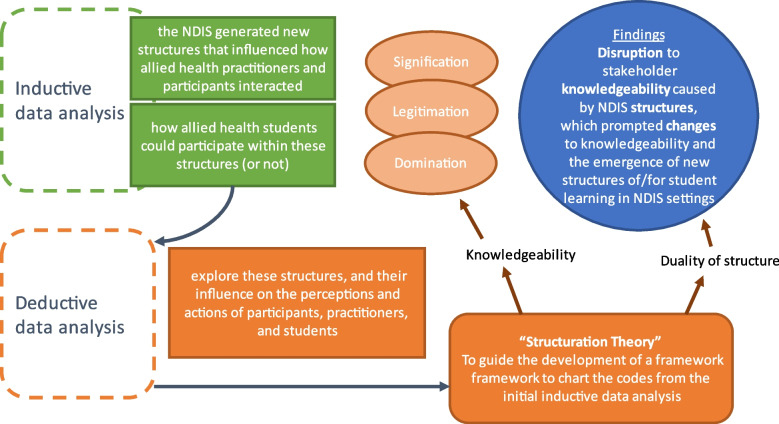
Data analysis processes

The first inductive analysis was conducted to inform the design of a conceptual model for allied health workforce design to respond to the NDIS drivers [5]. This analysis identified themes that described the impact of the NDIS on how allied health practitioners worked, and their changed role and relationship with clients. However, a prominent theme also related to how new NDIS structures impacted student education activities, and the opportunities and barriers to student placements within this context. As this suggested an interplay between the agency of the allied health practitioners to provide student education with introduced NDIS structures, a sequential deductive analysis of the dataset was conducted [29, 30] to explore the nature of these structures, and their influence on the perceptions and actions of participants, practitioners and students who are each stakeholders in the NDIS system. The sequential analysis was conducted by author 3 and reviewed by authors 1 and 2. Three concepts from Structuration Theory [23] were used to identify and define the codes: *knowledgeability; context in relation to space and time; and duality of structure* [23]. This informed a working analytical framework to chart the codes from the earlier inductive analysis (see Fig. 1, and refer to [5]). This process developed categories associated with the Structuration Theory codes that were then further sorted and refined to form early themes. However, data coded to the *context in relation to space and time* concept were identified as redundant when compared with the prominent themes identified from the remaining two concepts, and were collapsed into these to deepen the interpretation. The research team then further theoretically interpreted the meanings to identify and agree on final themes that reflected a rich exploration of the research questions.

### Rigour

The research team took field notes and memos during data gathering that further informed analysis. These were triangulated with data from the different stakeholder groups using a constant comparison approach to ensure that the themes identified were grounded in the data. Additionally, the ten participants who contributed two interviews provided data at progressive points in the project that enabled the research team to explore how their perspectives had adjusted as the transition to NDIS services unfolded, and to compare this with other data gathered across the project. Finally, the PAG provided feedback about the project findings and interpretation, and verified that the themes reflected their experiences and contexts. These processes assisted to interrogate the findings and assumptions, and the combined inductive and deductive inquiries enabled the findings to be audited to ensure that key ideas in the data had not been missed [30, 31].

The research team used project meetings to reflect about how their respective understandings of disability service provision, policy, allied health education and workforce development contributed to data interpretation and examined the biases that these presented. Researchers compared these with the knowledge generated from the stakeholder participants to ensure that the interpretation was congruent with the stakeholders’ realities [32].

### Findings

The findings describe how practice placements and student learning activities are understood, legitimised and prioritised in NDIS service settings as the stakeholder groups negotiated their time and space transition from the ‘old’ government-led funding to ‘new’ NDIS funding. The findings are reported according to the Structuration Theory concepts of *knowledgeability; and duality of structure* [23]. Necessary preface is presented in each section to contextualise the findings with the theory.

### Knowledgeability

The system of student placements enacted through NDIS service contexts includes three main stakeholder groups: (1) allied health students, (2) practitioner supervisors and (3) the NDIS participants. Each have agency within this triad and their own *‘knowledgeability’* about the dual objectives of participating in a disability service and student learning. When students complete a placement, their actions, those of their supervisors and the participants are guided by structures that inform their respective *‘knowledgeability’* of typical practices, participant behaviours and expectations, in addition to their own learning needs. Giddens proposed that knowledgeability is stored as three types of memory trace that direct how individuals understand and anticipate potential actions and outcomes, but which are bounded by the limits of their knowledge, prior experiences and the service context. How students, practitioners and participants use their knowledgeability in NDIS services may be interpreted using these memory traces – and these form analytical units to explore the findings: (a) signification, (b) domination and (c) legitimation.

- Signification

The first memory trace, signification refers to language concepts and semantic codes that provide structures from which the NDIS stakeholders make meaning. Signification structures direct how each individual understands service provision, the NDIS system and student learning, as well as how communication between students, practitioner supervisors and participants during the placement is interpreted.

Service providers understood quality placement as intrinsically related to how students interpret the NDIS principles and concepts:

*Quality placement is about being able to get an understanding of the NDIS and the implications of the NDIS context for service provision into the future … A placement that supports students to understand the importance of the important principles of the NDIS, so being person centred. Ensuring that they are supporting choice and control. (Adv 1).*

Supervisors and students reported differing understandings of what a quality placement meant, and actions that facilitate positive learning outcomes. For example, service providers perceived that quality placements required them to invest additional support and time to optimise students’ learning:

*Students do want a good quality experience. That requires additional support to make that happen, and the clinic needs to be able to find a way to provide those two things, but with … probably a heavier workload from the clinician that’s looking after those students to facilitate that and less capacity to earn from it. (Admin 2).*

Conversely, students understood quality placements to relate to their relationship and interactions with their supervisor:

*I had a really trusting relationship with my [supervisor] and as the placement went on, [I] became more independent and I really liked that feeling of independence because it really proved to me that I was ready and I was competent to work …*. *So I guess … if you don’t build a good relationship with your [supervisor], it can completely ruin your placement. (Student 9).*

For all stakeholders, the concept of participant ‘choice and control’ influenced how student placement activity was understood, and in turn, this placed boundaries around how student activities could be enacted. For example, some participants chose not to use their funds to engage students, as they understood students as having limited practice capabilities or were concerned about the equivalence of student and practitioner-led services. In the below quote, Participant 1 identified boundaries around the nature of student-led activities that they were prepared to engage with:

*I think probably [for] physio I would be a little bit more concerned. If they were doing the same sort of thing where … they’re just taking the lead. Because I think physio there can be a lot of damage that you can do if they’re not doing it quite right … (Participant 1).*

Similarly, many supervisors understood that participants perceive students as limited in capacity to provide quality services. Within a marketised environment, this generated tension for service providers who balanced providing attractive services in competition with other providers, with the benefits of student activity.

*… .. for example a family that I used to work with, they appeared to see the service as more of their right and they had very, very high expectations of clinicians - established clinicians, not just myself, and that family in particular I can’t imagine coping with having a student or accepting that. (Service Provider 2).*

Each stakeholder group understood that developing shared meaning about the nature of student activities and what supervision means was a facilitator of placement success. In the below example, the service provider recognised the importance of building meaning about the dual benefit for the participant and the student:

*Promoting the family’s understanding as to how the placements work and that it’s not a student just taking over the client, that there is a lot of support that goes into supporting a student. Having some of those fact sheets to give to families to promote their understanding of the student and how they’re involved with the NDIS and how that all works. (Service Provider 11).*

b) Legitimation

The second memory trace, legitimation, guides how individuals internalise rules, rituals and procedures. These structures enable students, practitioners and participants to understand and predict how a placement operates, and how student activities contribute to outcomes for each stakeholder. Service providers in this study were transitioning from the old, known government-led funding systems where how student activities ‘fit’ within structures was known and practised; to new service systems which required changed roles and routines. Practitioners reported high cognitive load from enacting these adjustments, and insecurity about their new positions and actions in these systems. They identified student placement activities as additional load that was difficult to accommodate within an already high-stress work environment.

*It’s just the growth and the set-up of having a new system, something that’s hard. If … we were more settled in and knew exactly what was happening and how it worked and it was consistent in the way it worked, it’d be easier to get the students to feel more comfortable with working. (Admin 2).*

‘Old’ understandings and processes about placements were superimposed onto emerging structures for NDIS service provision. For example, student placements often reflected traditional processes and routines as they were understood by students and supervisors, including time-tabling and placement activity scheduling; supervisory planning; and student assessment work. These legitimised structures were difficult to modify to accommodate new NDIS routines and processes.

*Then the learning opportunities were also at risk because it was service providers trying to navigate the NDIS world, but trying to do placements how they’d always done them. It was the challenge of, I guess, billable hours, KPIs (key performance indicators) for the therapists, new ways of having to think about service delivery, and then fitting students in. I guess the models or the resources and the work … actually need to consider the students as part of your business, as opposed to a separate offering. (Uni AHP Staff 1).*

Many service providers identified that these ‘old’ placement norms and routines were incompatible with new expectations of NDIS services. Placements were understood to require time-bound, legitimised student learning activities, including planning, assessment, feedback and reflection activities that scaffold student competency development. These recursive structures that are deeply embedded in the social and cultural practices of students and supervisors were often disrupted in NDIS service models, resulting in these stakeholders feeling insecure about how learning processes should unfold.

*They’re in a superior role. They are my supervisor. They don’t need to be my best friend, but they do need to … provide me with some support along the way that gives me some indication of how I’m travelling. I don’t want to wait for … evaluation to find out how I’m going. (Student 8).*

Service providers perceived that new NDIS structures which prioritised direct, billable services, left insufficient time to complete these expected placement activities. The legitimation of billable activities over other service quality and professional development activities disincentivised student placements.

*I’ve got a bottom line I need to reach. So if the time for students encroached on my billables, I think we would be less inclined to take students. (Service Provider 4).*

Progressively, some stakeholders identified opportunities to design new student learning processes to accommodate NDIS structures. For example, they conceived new structures related to quality service provision, student learning and billable activities. These new structures emerged across time and space, as stakeholders developed confidence operating within new service models, and where interactions between service and placement structures generated new understandings and routines. In the examples below, the service providers identified that student-led services could add value by lengthening sessions beyond the practitioner’s funded time, and through increasing the number of sessions available for participants. In both instances, students added value to the service by enhancing the participant experience and outcomes.

*The nuts and bolts of service would be delivered by the clinicians on the ground, but then there’s potential for extended [service] in terms of time and possibly depth …. Whereas there simply isn’t time allocated for a half hour of sensory [stimulation intervention]. (Service Provider 9).*

*You can have a clinician in the room, which we had done in our placement. So, [clinicians are] in the room with our students so then it’s the full rate. However, there is an opportunity to do, say, an extra two sessions at a cheaper rate if the clinician’s not in the room. (Service Provider 11).*

Similarly, placement structures evolved to contain student learning activities that could be accommodated within new NDIS service structures.

*We did a lot of written feedback; so, getting them to write down strengths, challenges, things they did differently. I created a bit of a template and got them to do that, working towards their competencies... Also, a lot of the online feedback, particularly driving between clients, feedback in the car, debriefing straight after, problem solving through anything that happened in the session was able to be done in the car. Also, setting time aside on a Wednesday afternoon to have a chat about how things are going, how they’re feeling, reflecting. (Service Provider 11).*

However, these new structures also modified expectations about the role of students as learners, as billable activities became more visible in placement models. Many students perceived a tension between their status as novice practitioners with learning needs, in contrast to new expectations of their capabilities to provide services for paying participants.

*It was just the fact that the parents had been told that I would be supervised and that was part of the explanation for them being charged a full rate and that’s what kind of made me feel a little bit uncomfortable. (Student 4).*

iii) Domination

The final memory trace, domination, captures understandings about how power and resources are allocated, both in relation to resources that facilitate placements, and within the triadic relationship of the student, practitioner supervisor and the NDIS participant.

In new NDIS structures, service providers understood that resources for non-billable activities needed to be distributed across many quality activities, including student learning. This prompted decision-making about how best to allocate resources for the best benefit of the organisation, practitioners and participants.

*You’ve got a certain amount of space and a certain amount of resource that we need to utilise. If we’re taking a chunk of that out to put into students, we’re also removing that chunk and that capacity from our practice (Service Provider 2).*

In traditional placement structures, the triadic relationship enabled the practitioner to select and moderate the work that students were permitted to complete, and the nature of services that students provided. Reflecting about how the student supervision relationship has changed as a result of NDIS reform, a service provider observed:

*The trick has always been the student has to be the one working with the client. But in some situations that’s … impossible, particularly for the first part of their placements. These people: there are all nuances and skills that come into [practice with them]. Having a student, and us say, “right, on the fourth visit I’m going to let you do the session, like I did when I was a student” …. I don’t see that model working very well in our setting. (Service Provider 7).*

New structures premised on participant choice and control increased the power of participants to direct when, how and where their services were enacted in accordance with their preferences and whether to engage students in their service provision. Participants often preferred services delivered within their own homes or local community settings, in times and with methods individualised to their needs. However, service providers perceived these as more challenging contexts to accommodate student learning.

*The logistics makes it hard, so … desk space, or whether they’re travelling to clients all the time and then if you’re taking four students out to see one participant … Four students can’t go into that house or even two sometimes is too hard to go into that house, so what do you do with the other student and what do you get them to do? We tried to work through these difficulties with service providers, but that’s still … a huge challenge from their perspective about having students. (Uni AHP Staff 2).*

New NDIS structures also adjusted the practitioner’s power to determine how they deliver participant services, operate in their organisational work environment, and supervise students. The practitioners’ uncertainty with these organisational and practice structures also constrained their capacity to identify new processes to support student learning.

*The organisations that were predominantly block grant funded they’re really struggling because it’s in a completely different mindset. The culture change that those organisations are having to go through is huge …. It’s changing that … operating model and changing a person’s own view of the value of their work, which is really - a lot of people are really struggling with it … It’s probably been the most common reason for people leaving the sector - … cognitive dissonance. (Adv2).*

For students, this shift in the position of their supervisor within the triadic supervision structure and in service provision also changed their ascribed role and routines as learners on placement. Supervising practitioners often responded to their uncertainty in new service structures by either increasing or reducing their control over the students’ activities. Consequently, some students reported that supervision activities were heightened or absent in NDIS placements.

*I feel like, for me personally, I’ve been kind of restricted and choked … I just feel like … I’m not being the clinician - I feel like I’ve always got someone watching over my shoulder … (Student 9).*

*Going into a session, a lot of anxiety. With parents, no supervision, with a client I’d met once and knowing they’re paying full rate. (Student 3).*

However, when practitioners brokered a shared understanding of how students could add value for participants, and the roles of each person in the triad were negotiated and more explicit in the context of the NDIS service, these power imbalances reduced.

*That makes a better working relationship with the student and the client. So … the clients don’t come in feeling I’ve got a student again today, they’ve actually made that choice. I think then that builds a better relationship with the client and the student. (Service Provider 9).*

### Duality of structure

Giddens identified an interdependency in the relationship between individual’s actions and structure, where each influence and modify the other across space and time: the *‘duality of structure’*. With respect to the NDIS context, new placement opportunities emerged over time, reflecting a maturing understanding of how student placement activities could benefit all stakeholders. New structures reflected a synergism between the NDIS participant’s chosen goals and activities, and student learning needs. Supervisors, participants and students reflexively monitored and adjusted how students contributed to the participants’ goals and service provision.

*We’ve been doing a lot of work over the last 12 months in terms of looking at how we offer student placements to work within a private practice setting such as ours and with … due consideration to the NDIS … We’ve done a bit of talking with … the University about that model and how it works, and we’ve gone from having just two students to having groups of four students. (Admin 2).*

New placement structures were trialled and adjusted over time to optimise student learning opportunities through responding to participant needs. In this way, it was the actions of participants, service providers and the students themselves that modified the traditional structures for placement, and the understanding of how student learning unfolded in placements.

*I think it (having students) is a huge benefit for pretty much everybody. A student has a lot more time to be very specific and individualised to a client … They’ve got a lot of time to make the [… board game] that is specific to that child, which is going to increase his engagement, which [for] me running a full caseload … I don’t have time to make those individualised … reinforcements for things like that. (Service Provider 8).*

As service providers monitored how these new placement configurations met or enhanced practice and participant outcomes, they revised their knowledgeability and worked to expand these practices to benefit a wider range of stakeholders.

*If we were to look at having OT students as well and setting up some environments in which the speech and the OT students could do a proportion of their work together while they’re on placements … I think that that would be really useful because I think that the OT students and the speech [pathology] students would be able to support each other’s learning, just like our speech students support each other’s learning as well. (Admin 2).*

To respond to the increased power of NDIS participants in the placement system, supervisors adjusted their role to include negotiating with participants regarding how student-delivered services could meet their needs and goals. The resulting legitimation structures enabled supervisors to broker the participants’ understanding of the potential benefits. This changed the meanings that participants ascribed to engaging with students as part of their service, and increased their likelihood of making this choice.

*I think … with the NDIS and with the funding …. a lot of the families, once it was really explained, were really on board and really supportive of having a student … It’s just building that at the beginning was really, really important to the success of the interactions and student placements. (Service Provider 11).*

As new practices became recursive, the benefits for all stakeholders became more visible in the system. For example, new ways of construing student learning prompted new ways of understanding the learning relationship between NDIS participants and students. This, in turn, unmasked new ways for participants to target their NDIS goals that had been less visible in traditional placement structures.

*I think the families teach the students so much. I think your clinical skills are important but actually your relationship building skills, your ability to empathise and get people on board, see the bigger picture is really what families are teaching the students. (Service Provider 14).*

Similarly, workforce development benefits for the service providers themselves became more visible as structures emerged that combined benefit for participant and student learning outcomes.

*I think we’ve looked at a model to work for us and make it sustainable and how … by putting the right supervision and mentoring in place they’re more likely to stay in disability - at least the sector... Because we’re trying to attract a talent pool. Even if they don’t stay with me we’ve got to attract … a talent pool so that at least I access them at some point. (Admin 3).*

### Summary

**Fig. 2:**
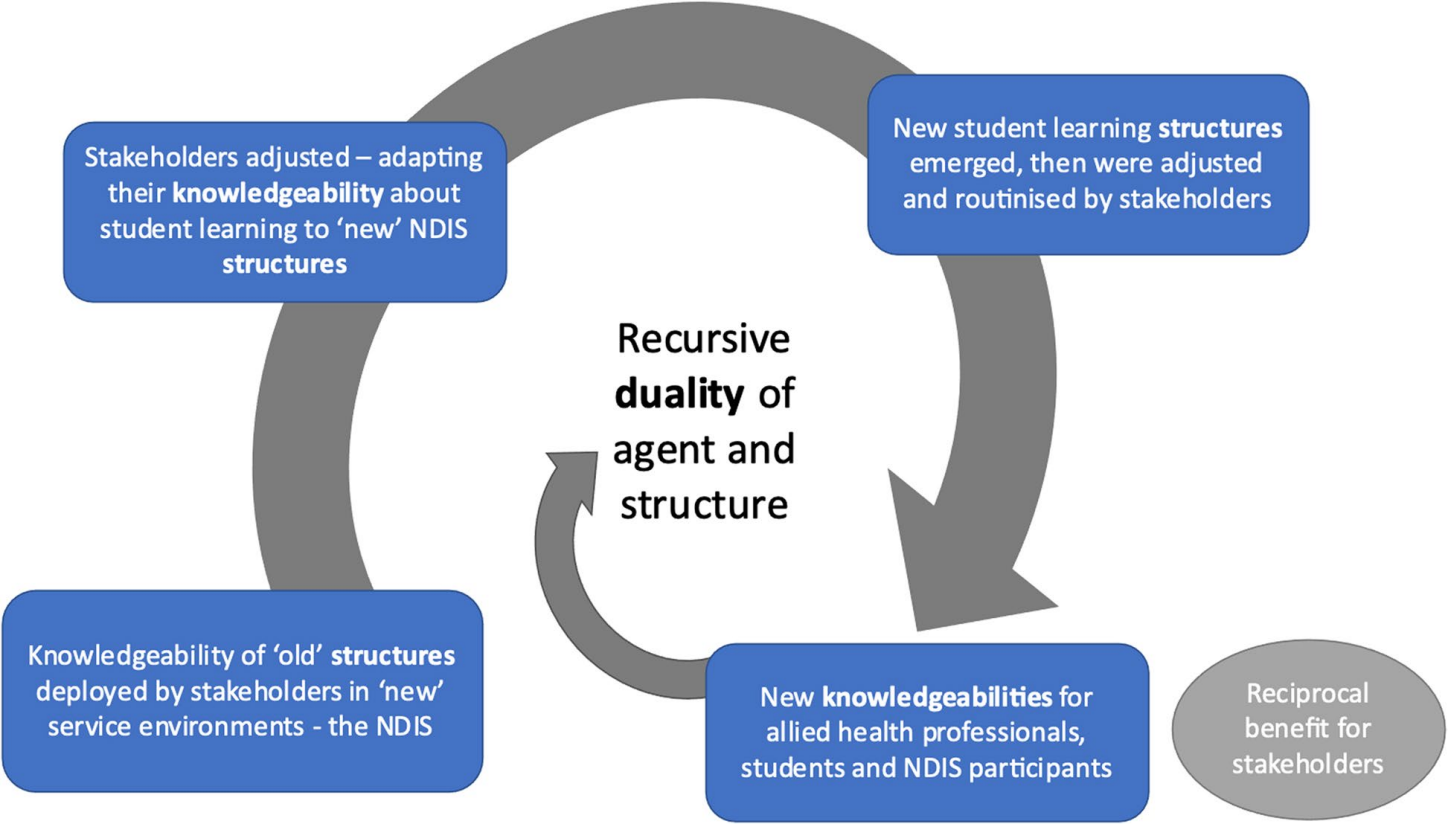
Emergence of new NDIS placement structures over time

Student learning in placements has been guided by traditional, recursive structures that are bounded by the knowledgeability of students, service providers and participants. The transition to the NDIS resulted in a disrupted service provision environment where old placement structures – and stakeholders’ knowledgeability – could not respond to NDIS participants’ chosen goals and activities. However, over time and in response to the contextual features of the service and participants, new placement and student learning configurations were identified, monitored and adjusted to respond to the needs of all stakeholders. Transformative NDIS concepts, such as ‘billability’ and ‘participant choice and control’ were interpreted to fit across the Structuration concepts, and the emerging structures can be understood as broadly impacting on allied health and student education practices. As changed structures became routinized, they influenced the knowledgeability and actions of each stakeholder group. New student learning and placement structures were progressively refined for the needs of service providers and participants, and optimised for student learning and workforce development opportunities. Figure 2 illustrates the iterative adjustments, enacted by stakeholder groups, that evolved and refined new structures to facilitate student placements in the NDIS system.

## Discussion

Placement activities for allied health student learning and workforce development in the newly marketised NDIS service context can be usefully conceptualised through Structuration Theory. Analysis guided by Structuration Theory draws attention to the cognitive structures stakeholders hold and enact to navigate social life. Giddens observed that interactions within social systems are situated within time, space and contexts that themselves move, transition and change. Stakeholders in this study reported uncertainty in how to interact in new NDIS systems that were transitioning from government-led to participant-led funding. As service providers routinised new business-oriented structures, they were progressively more able to introduce placement activities that facilitated students’ capability to provide NDIS services as a workforce development strategy. However, our findings suggest that tensions exist between the new NDIS service models and student learning expectations and behaviours.

Through the early formative period of the NDIS transition, study participants identified that new service provision models were not compatible with legitimised structures and expectations that had previously guided allied health student placements. Giddens noted that individuals are directed by largely internalised social structures that inform the ways they act and what they understand of the systems they operate within. The meanings, traditions, routines and power relationships that the service providers, students and NDIS participants expected to guide placement activities had been developed in historical practice structures that were radically shifted in response to the funding reform. The reported barriers to NDIS student placements often reflected an attempt to superimpose old, known student placement activities, without adjusting for new structures that empower the NDIS participant and provide greater choice and control in their services and outcomes. For example, the changed domination structures within the traditional triadic practice education relationship [22] shifted power from the supervisor, affording greater power to the participant. Supervision strategies that had previously scaffolded students’ learning through progressive exposure to participants and practice [33], and gradually entrusted students with more complex practice opportunities [34] needed to be re-conceptualised for the participant-led nature of the NDIS system.

This research found that establishing shared meaning and the mutual benefits of engaging in placement activities for all stakeholders were facilitators to successful NDIS student placements. However, in reality, new placement structures often aligned with value-adding activities for participants and service providers that were less attentive to students’ learning needs. In this vein, the emerging NDIS structures influenced how each stakeholder group understood the role of students and their activities as directly contributing to service provision, rather than learning in the milieu of the workplace [14, 33]. New signification structures that ascribe student activities as contributing to service provision may be in tension with the understood purpose of placement as facilitating students’ competency development and being learner-oriented [14, 15, 22]. This subtle reorientation assumes that student learning is a product of providing participant services, but this may diminish the meaning and contribution of intentional educational activities in placement such as modelling, scaffolding, feedback and reflection [19] that are less congruent with funded activities in participant-led services. Whilst new placement structures legitimised student activities as part of NDIS service provision, both students and service provider participants in this study identified challenges in affording an effective learning experience for students.

While the NDIS reform has transformed organisational and service provision contexts, the pedagogy, learning expectations and intended outcomes of practice placements for allied health students have remained fundamentally unchanged. The tension identified in this study, in how legitimised learning and practice assumptions and processes that underpin student placements translate to newly emerging participant-led service provision models, have not been explored in health professional education research. For example, allied health practice education is grounded in learning theories [35] that inform placement learning activities such as scaffolded skill development (e.g. [36]), goal-setting, modelling and part-task activities (e.g. [37]), feedback and reflection (e.g. [38]). Whilst practitioner time was often allocated to support such student learning activities in historical services, participant-led funding that is increasingly common to disability, health, aged care and education sectors require that these activities align more directly to billable participant services to minimise financial implications for host service providers. More research is needed to identify what and how students learn in these leaner service models, and how this learning aligns with and supports students’ professional competency development. Universities have an important role in co-designing contemporary student placement structures with service providers, and particularly in response to transformative policy reform such as has been demonstrated in participant-led funding, illustrated in this study through the NDIS. Student learning and placement activities more broadly risk becoming redundant or burdensome to organisations and practitioners if universities are unable to monitor and adjust learning structures and expectations in response to sectoral and professional practice change.

## Limitations

Structuration Theory is a practice theory often applied to explore activities that unfold, are produced and then become reproduced within a particular social context [23]. Our data were gathered and analysed within the particular social and time-bound context of the NDIS transition in South Australia. The social practices and structures that relate to NDIS service providers who did not participate in our study, or who operate in non-NDIS participant-led funding environments will differ to those included in this study. As such, the learning from this study should be translated cautiously, as student activities need to be modelled sensitively to local social and service provision contexts – which emerge in particular time/space interactions.

For this study, interview data gathered iteratively across the project was analysed as a single dataset to understand how stakeholders were operating within the marketised environment. An iterative approach to data analysis that tracked emerging service and practices across the NDIS transition period may have provided useful additional information about how student placement and learning structures were adapted over time. This missed opportunity to explore how structures adjusted across the period of critical implementation period may limit how this research could apply to future policy reform.

## Conclusions

Participant-led funding is intended to invoke changes in organisational systems, processes and practices that respond to consumer behaviours and market trends. These structural changes transform how participants, practitioners and other stakeholders understand the nature and purpose of services, and the ascribed power and position of participants within the new service landscape. In concert with these shifting structures, this study explored the meanings and practices given to allied health student placement activities, that relate to workforce development and quality that are less explicitly identified in the funding model, but that must necessarily adjust to the market environment. With emerging participant-led service provision models, there is a need to ensure that student learning activities in the workplace, as a key workforce development strategy, are compatible with new services, manageable for supervising practitioners, and meet the service needs and priorities of participants. This study identified that whilst service providers were able to develop new placement structures that met NDIS participants’ needs over time, tensions remain in how the system can facilitate student learning and competency development. New service provision systems require further conceptualisation to ensure that the pedagogical and practice structures needed to assure quality allied health student placements are able to complement the service goals and needs of NDIS participants.

## Supplementary Information

**Additional file 1.**

**Additional file 2.**

**Additional file 3.**

**Additional file 4.**

**Additional file 5.**

**Additional file 6.**

**Additional file 7.**

## Data Availability

Please contact the corresponding author for any queries related to the data and materials utilised to inform this study.
