# Supplementary material for: Allied health workforce development for participant-led services: structures for student placements in the National Disability Insurance Scheme
Source: BMC Med Educ. 2023 Feb 6;23:95. doi: 10.1186/s12909-023-04065-y (PMC9903456; doi:10.1186/s12909-023-04065-y)
Supplement: Supplementary file 1 — Additional file 1. [file 12909_2023_4065_MOESM1_ESM.docx]

**Interview Schedule for Early Implementation Issues**

Preamble:

*Firstly, thanks for your participation and hard work in the project so far.*

*The project is on track and we are coming to a close in June. We’re trying to get feedback from a wide range of stakeholders about the opportunities and challenges for student placements which occur in NDIS settings.*

*We’re particularly interested in your thoughts of which student placement models (i.e. mentoring, multi-student, 1-1 etc) work well in which settings that handle NDIS funding. We would also like to understand the complexity of maintaining a quality service for clients and a quality learning opportunity for students and what kind of new challenges the NDIS throws up for universities and placement coordination.*

*That being said, of course there’s a whole range of other topics that may be relevant for us to discuss. Some of these we will explore in more depth in later interviews during the project, so any and all of the insight or suggestions you have about the student placements will contribute to the ongoing design and adaptation of these models.*

*Sign consent form x2*

**Interview Questions**

Can we start by you telling me a little bit about how you think the trial placements at services you’ve been involved with are going?

Can you tell me a bit about what the billable activities that you’re aware students are involved with?

Has the billing of services be raised explicitly as either a barrier or an issue for service providers taking on student placements?

The NDIS talks about students value-adding to practices. In what ways do you see students value-adding to the client experience?

What about value-adding to the service?

So we’ve really been talking about financial viability. Do you have any further comments on this issue in relation to student placements within the NDIS funding model?

*Show model if face to face or send if phone interview*

These were thought to be the non-negotiables at the start of the project. How do you think these principles have translated into the trial placements you’ve seen running throughout the project?

In wearing your hat as a placement coordinator, can you tell me about the quality of student placements (in terms of student learning) that have been running as part of the placement? Do you think the factors that influences the quality of student placements are represented in the model?

In the project so far, we’ve noted that there’s been a bit of an expectation that students hit the ground running. Have you experienced this? What are your views on this?

Do you think there’s a difference in the teaching learning relationship in an NDIS funded service, rather than placements under block funding?

We’ve got some people that are taking multiple students, and some that take singles, some that run a peer mentoring program… From your observations, what are the outcomes in terms of teaching or learning?

Do you think students are being exposed to multidisciplinary and interagency work within their placements in your organisation? Have you had any experience with multidisciplinary supervision? Has that worked? What have been challenges to this in the context of NDIS funding? What is your knowledge on how services are able to bill for this multidisciplinary work?

We know you’ve been working closely with the placement facilitators. In your view, how has this interacted with site capacity to host student placements?

In your view, what do you think services benefitted from the most? Is this different to what services describe being most useful about the placement facilitators?

Does the NDIS model throw up particular challenges for the universities?

How do you think universities can support student placements which occur with NDIS funding?

Is there anything I haven’t asked so far that you think I should have? Or, do you have any other comments?
