# Supplementary material for: Allied health workforce development for participant-led services: structures for student placements in the National Disability Insurance Scheme
Source: BMC Med Educ. 2023 Feb 6;23:95. doi: 10.1186/s12909-023-04065-y (PMC9903456; doi:10.1186/s12909-023-04065-y)
Supplement: Supplementary file 2 — Additional file 2. [file 12909_2023_4065_MOESM2_ESM.docx]

**Interview Schedule for Early Implementation Issues**

Preamble:

*Firstly, thanks for your participation and hard work in the project so far in your role as a placement facilitator.*

*This interview is an opportunity for the project team to gain feedback about the models for student placement which you have been involved in implementing with service providers so far.*

*We are interested in your experiences with students and placements delivered during the first phase of the project. Any insight or suggestions you have will contribute to the ongoing design and adaptation of these models. I have a short list of topics to discuss, but we recognize that each stakeholder has a particularly unique viewpoint, so if there is something missed please don’t hesitate to let me know.*

*Sign consent form x2*

**Interview Questions**

Thanks for being willing to be interviewed as part of the project. Could we start by you telling me how you describe your position to new sites or stakeholders?

What do you see as the most important part of your role?

Which placement sites are you involved with, and what kind of models they have running at the moment?

In your experience, are there really clear model/s indicated for specific placement sites? Or, can the dimensions of student placements be adapted to make every placement model in any setting?

- Setting (metro/rural, sector, NGO/PP, multi/inter/trans-D)
- Timing (calendar, freq, duration, flexibility)
- Practice population (their characteristics)
- Transition (handover, overlap)
- Orientation (NDIS, org)
- Student (yr level, capability, Masters/Bach, learning trajectory)
- Supervision (mentoring, peer support, single/multiple students, shared supervision, cross-D supervision, remote supervision, university educators)
- University (communication, support)

Are any of the dimensions more critical to informing what placement model should be undertaken than others?

Can you paint a picture of the sort of interactions you have had with various sites? What do you think have been some of the more significant developments you have been involved in (e.g. problem-solving re a particular placement, suggesting innovative models, providing ‘just in time support”)

From your perspective, what are the benefits of the placement facilitator role in support student placements? Can you give some examples?

What are some of the key challenges of your role? Can you give some examples?

Do you feel confident that you know how to advise services to bill for the various ways of facilitating of a student placement, including all the activities associated with the clinician’s time? Is there anything that you are still uncertain about in terms of pricing and compliance?

*Need to note specific +concrete ideas here (i.e. who, what, where, when, how) to be able to present to NDIA + get feedback on; i.e. ‘under these circumstances this is how service providers are billing their clients for that time.’*

Do you have any comments on the financial viability of any of the placement models, within NDIS funding? Can you tell me about them?

Regarding all the contact you’ve had with various stakeholder groups as part of the project, what kind of working relationships do you think are most effective?

To what extent do you think that high-quality student placements are feasible in services which offer NDIS funding? What do you see as being most critical to this outcome?

If a service provider was adamant that they were concerned about taking on students because of their workload, what would you say? Would you have any advice for them?

If you put on your ‘clinician’ hat as opposed to your ‘placement facilitator’ hat, would you give a different answer?

Could you see a placement facilitator role being sustainable beyond the life of the project? Would you make any changes?

Is there anything I haven’t asked so far that you think I should have? Or, do you have any other comments?
