# Supplementary material for: Allied health workforce development for participant-led services: structures for student placements in the National Disability Insurance Scheme
Source: BMC Med Educ. 2023 Feb 6;23:95. doi: 10.1186/s12909-023-04065-y (PMC9903456; doi:10.1186/s12909-023-04065-y)
Supplement: Supplementary file 3 — Additional file 3. [file 12909_2023_4065_MOESM3_ESM.docx]

**Interview Schedule (semi-structured; ~30-45 mins):**

*NDIS Recipients*

*Preamble: Firstly, thanks for taking the time to meet with us today. As you would know the NDIS is leading to lots of changes in how services are provided for people with disability. One of those things is how we provide placement-based training for allied health students so that they are well-equipped to work with people who have disability when they finish their degree. That’s what this project in particular is exploring.*

*I’m going to ask you to sign this consent form. It says that you don’t have to answer any questions you don’t want to, or ask to end the interview at any time. If you need to do either of these things there will be no negative consequence, either today or on behalf of the service provider who referred you on to us.*

*The interview will be recorded only so we can make a typed-up version of what you say, to help us with looking at the results later. When it’s typed up your name is removed so what you have shared isn’t linked to you anymore. If we use any of the information you provide won’t be published next to your name, however, some people may be able to gather it’s come from you.*

*Do you have any questions about any of these things? Do you feel like you understand everything that’s in the consent form, and that you’re happy to sign it?*

**Get consent form signed x2; they keep information sheet + 1x consent form**

*The questions I’m going to ask you are about how NDIS participants might feel about having students in training as part of their service provision. There are no right or wrong answers. At the end of the interview we’d just like to know what you think is important for universities and service providers to know when thinking about how to best-train students in the context of the NDIS. If anything’s unclear, please feel free to ask questions as we go along.*

Start of questions

So perhaps we could start with a bit about your involvement with the NDIS. Has it rolled out to your region/age group yet?

Has it changed your experience of service provision, and if so can you tell me a bit about how?

As part of services that you receive, have you ever been involved with students before?

Questions about students (if they’ve had them)

What did that look like?

How did you first feel about it?

Some people say it can make them feel a bit nervous about it, especially the first time. Was there anything that made you feel more confident about it?

How did your expectations compare to what it was like? What were good parts, and what were not-so-good parts?

Do you think anything could have been different, to make your experience with students better? What suggestions would you make?

Questions about students (if they’ve **not** had them)

If your service provider asked you if they could bring students along to sessions so they could learn and observe, how would you respond?

What if they asked if students could provide a service to you with direct supervision – i.e. the therapist being in the room?

How about if it was only the student that came to visit, and the therapist provided planning and debrief support to them?

Would there be any services or activities to which you would say ‘definitely not’?

Are there any characteristics of participants which might change your answer to any of those questions?

Questions about students in the NDIS

One of the possibilities that’s being considered is using student services to ‘extend’ or ‘add value’ to packages in the NDIS. This boils down to the actual student service being free, but the supervision provided by the clinician being billed to the client, so possibly services would be cheaper altogether for participants. What are your first thoughts on this?

Would you see any safety issues or other challenges for how students could be involved with offering services to service recipients, either yourself or others? What would these be?

Do you have any ideas for how students could be involved in service provision?

Are there any services you wish you could be funded for but aren’t? Could students help with that somehow?

If a friend said that they were unsure whether or not to agree to having students as part of their service provision, what advice would you give them?

If you were trying to persuade a friend it was a good idea to have students providing services, what kind of benefits would you promote?

When you receive your plan, what are the kinds of information you use to decide about which service provider to go to? Would a service provider who advertises a commitment to training students be a positive or negative? Would it depend on other information, like how they plan to use students?
