# Supplementary material for: Allied health workforce development for participant-led services: structures for student placements in the National Disability Insurance Scheme
Source: BMC Med Educ. 2023 Feb 6;23:95. doi: 10.1186/s12909-023-04065-y (PMC9903456; doi:10.1186/s12909-023-04065-y)
Supplement: Supplementary file 4 — Additional file 4. [file 12909_2023_4065_MOESM4_ESM.docx]

**Project Script:**

Thank you for making time to participate in this interview which will explore the trial student placement models involving NDIS participants. This study is part of the project entitled *Building an Allied Health Workforce for NDIS Funded Service Delivery* being undertaken by Flinders University researchers in partnership with the Department of State Development. We aim to interview people with varying perspectives on the trial: policy-makers, practitioners and managers, educators, students and NDIS recipients.

The interview aims to gain information about the barriers and enablers for student involvement in NDIS services and your experience of this and any suggestions for the future. The sharing of your experiences will assist in the development of student involvement in NDIS funded services. Such experiences are important in developing allied health professionals with the skills, knowledge and values to partner effectively with NDIS participants.

We anticipate the interview will take approximately 30 minutes. If you would like any further information or clarification please contact myself or the chief investigator. The study has ethics approval from the Flinders University ethics committee.

**Interview Schedule:**

Can you tell me about your role and how it relates to the NDIS rollout?

Have you had any experience with student placements in disability settings so far?

*Prompt: focus on student placements not NDIS rollout*

Have any student placements you’ve seen been particularly innovative, or had to change to adapt to changing circumstances?

*Prompt: what made them successful?*

What do you think some of the common traps are in collaborative student placement models?

*Prompt: underlying assumptions // conflicting expectations*

What do you see being the advantages and disadvantages for who’s involved?

*Prompt:*

- *Recipients*
- *Students*
- *Providers*
- *Organisation*
- *Profession*

Can you think of how placements could draw on multi-D or trans-D practice?

What can you see being an opportunity for student placements in the NDIS environment?

*Prompt:*

- *Individual or organisational*
- *Costs*
- *Agency/organisation*
- *NDIS recipients (nature and extent of disability/vulnerability)*
- *Practitioners*
- *Nature of work/safety (students and clients)*
- *Placement expectations and structures*
- *Support for supervisors/students*
- *Collaboration between universities and schools*
- *Collaboration between service providers (+ NDIA)*
- *Stages/sectors of rollout (i.e. different client groups)*
- *Relationship with health and education sectors?*
- *Values*

How about any challenges for students engaging with NDIS services and recipients?

- *Organisational energy is being directed into figuring out how the NDIS works and how to adjust to changes in how the scheme is delivered*

Can you think of any ideas that would help overcome those barriers?

*(prompts)*

- *Resources*
- *Processes*
- *Models*
- *Structures*
- *Values/Approaches*

The long-term goal is thinking about sustainable student placement opportunities in the NDIS context – what do you think the short and medium term goals need to be?

Do you think there are any specific service gaps in the NDIS – and could these be addressed somehow by student placement models?

Is there any other information or questions that you would like to add that can contribute to the relevance of this project?

Do you have other contacts who have experience/this kind of knowledge? Do you think they would be willing to be interviewed?
