# Supplementary material for: Allied health workforce development for participant-led services: structures for student placements in the National Disability Insurance Scheme
Source: BMC Med Educ. 2023 Feb 6;23:95. doi: 10.1186/s12909-023-04065-y (PMC9903456; doi:10.1186/s12909-023-04065-y)
Supplement: Supplementary file 5 — Additional file 5. [file 12909_2023_4065_MOESM5_ESM.docx]

**Interview Schedule for Early Implementation Issues**

Preamble:

*Firstly, thanks for your participation and hard work in the project so far.*

*The project is on track and we are now in phase two of the project, which involves seeking feedback about the models for student placement which have been implemented with service providers so far. We are interested in how you think the trial placements have gone within your service.*

*We’re particularly interested in understanding more about the business and billing considerations of having students on placement under NDIS funding. An outcome of our project is to be clear on how clinician, management and/or student time can be billed for under the NDIS. We’d like to make sure we have a concrete understanding of some of the issues from the perspective of clinicians and service providers so that we can work to clarify these by the conclusion of the project.*

*That being said, of course there’s a whole range of other topics that may be relevant for us to discuss. Some of these we will explore in more depth in later interviews during the project, so any and all of the insight or suggestions you have about the student placements will contribute to the ongoing design and adaptation of these models.*

*Sign consent form x2*

**Interview Questions**

*Italicised text = things for consideration by interviewer prior to arriving at interview, in order to make the most of interviewee time + insight*

Can we start by you telling me a little bit about how you think the trial placement at your service went?

*(open, inductive, not leading)*

*Combing through a bit more specifically with these next questions; needs some input by interviewers regarding the ‘dimensions of placement’ matrix put together by Nicole/Kendal.*

I’ve put together some questions based on the dimensions identified to be pertinent to the student placements hosted at your site.

*i.e. how is transport an issue (for students/clients/clinicians supervising students) in your Northern-based service?*

*i.e. do you think the peer support model that you used matched the needs of the client group well?*

*i.e. did the calendar timing of students work well for the clients you service? How about the duration of placements, or the need for flexibility of service provision (time of day/intensive blocks) for your clients?*

- Setting (metro/rural, sector, NGO/PP, multi/inter/trans-D)
- Timing (calendar, freq, duration, flexibility)
- Practice population (their characteristics)
- Transition (handover, overlap)
- Orientation (NDIS, org)
- Student (yr level, capability, Masters/Bach, learning trajectory)
- Supervision (mentoring, peer support, single/multiple students, shared supervision, cross-D supervision, remote supervision, university educators)
- University (communication, support)
  - *Keep in mind we also want to explore what an ongoing relationship strategy with the uni/s needs to look like outside of the scope of the placement facilitator, to ensure that these strategies are appropriate/sustainable in the future*

Can you tell me a bit about what the billable activities are that students are involved with at your service?

What about the activities that students do at your site, which aren’t NDIS funded? What are they?

What do you think the balance is between these two activities?

For the activities that aren’t billable, how would you see those activities adding value to the client?

What about adding value to the practice?

Obviously the time you are in a room with a student can be billed as supervision, but there are some things that are less tangible in terms of billability – for example, if I’m having a debriefing session with you about professional behaviours, that’s not ‘as’ billable. If you think about the total time that you provide supervision to students, to what extent are you able to claim that time back under billable activities?

Do you feel confident that you know how to bill for the various ways of facilitating of a student placement, including all the activities associated with the clinician’s time? Is there anything that you are still uncertain about in terms of pricing and compliance?

*Need to note specific +concrete ideas here (i.e. who, what, where, when, how) to be able to present to NDIA + get feedback on; i.e. ‘under these circumstances this is how service providers are billing their clients for that time.’*

Do you have any comments on the financial viability of this kind of placement, within the NDIS funding model?

*Show model if face to face or send if phone interview*

These were thought to be the non-negotiables at the start of the project. How do you think these principles have translated into the trial placements you’ve run?

Do you think students are being exposed to multidisciplinary and interagency work within their placements in your organisation?

Since we last interviewed you, the placement facilitators have come in to support student placements. What contact have you had with them?

In your view, what was useful and what wasn’t useful? What would you like more of/less of?

Thinking about your engagements with universities in the past, what are your perspectives on what does or doesn’t support student placements which occur under NDIS funding?

If another clinician told you they were concerned about taking on students because of their workload, what would you say? Would you have any advice for them?

*For principals + managers, can ask: if you put on your ‘managers hat’ where you’re concerned about finances, or if you put on your ‘clinician hat’ is there a difference in how you’d answer the question?*

Is there anything I haven’t asked so far that you think I should have? Or, do you have any other comments?
