# Supplementary material for: Allied health workforce development for participant-led services: structures for student placements in the National Disability Insurance Scheme
Source: BMC Med Educ. 2023 Feb 6;23:95. doi: 10.1186/s12909-023-04065-y (PMC9903456; doi:10.1186/s12909-023-04065-y)
Supplement: Supplementary file 6 — Additional file 6. [file 12909_2023_4065_MOESM6_ESM.docx]

**Interview Schedule for Final Interviews**

*Firstly, thanks for your participation and hard work in the project.*

*The project is wrapping up now and we are looking to explore your views on the project and the remaining issues. We are interested in how you think the trial placements have gone within your service and more broadly ongoing challenges for student placements in NDIS funded settings.*

*We’re particularly interested in this round, to understand what you think makes up quality student learning and how this is compromised or strengthened by various aspects of NDIS funding.*

*Sign consent form x2*

**Interview Questions**

One of the underpinning principles for this project is that the student placements provide quality for all of the stakeholders. By all we mean… Let’s start with you as a service provider. What do you think makes up a quality student placement in the NDIS context?

What about for the clients; what’s a quality experience for them? [prompts: care plan…]

What about for the students; what’s a quality experience for them? [prompts: approachability…]

As a service provider what do you think makes placements a quality educational experience for students?

Do you think there’s a difference in the teaching learning relationship in an NDIS funded service, rather than placements under block funding?

Under block funding, it was easy to involve student in groups, they might have been involved in prevention or promotion type funding. These have become more difficult during the NDIS. Do you think what students’ actual experience is on student placement changed under the guardrails of NDIS funding?

What activities are allied health students involved with at your site that you think are optimal for quality student learning?

There are all these different models of student placement and supervision. What have you had experience with – what does that particular model have to offer in terms of providing a quality experience for all the stakeholders?

Are there any trade-offs where amplifying quality for one stakeholder would compromise quality for another?

What role do you think NDIS recipients can play in teaching students on placement?

Do you think students are being exposed to multidisciplinary and interagency work within their placements in your organisation? Have you had any experience with multidisciplinary supervision? Has that worked? What have been challenges to this in the context of NDIS funding? What is your knowledge on how services are able to bill for this multidisciplinary work?

We know you’ve been working closely with the placement facilitators. What influence has this had on the site’s capacity to design and deliver student placements?

If you were giving advice to an organization who was considering to host student placements in the context of NDIS funding, what tips and tricks would you pass on to ensure they were successful?

Is there anything I haven’t asked so far that you think I should have? Or, do you have any other comments?

*Supplementary – ask if you have time!*

Does the NDIS model throw up particular challenges for your relationship with the university – service provider relationship in regards to facilitating student placements?

How do you think universities can support student placements which occur with NDIS funding?
