# Supplementary material for: Allied health workforce development for participant-led services: structures for student placements in the National Disability Insurance Scheme
Source: BMC Med Educ. 2023 Feb 6;23:95. doi: 10.1186/s12909-023-04065-y (PMC9903456; doi:10.1186/s12909-023-04065-y)
Supplement: Supplementary file 7 — Additional file 7. [file 12909_2023_4065_MOESM7_ESM.docx]

**Focus Group for Students**

3x separate groups of 8-10 students; for groups ~45 minutes

Objectives as stated in Project/Ethics Application:

- Ask a few questions about your experience of student placement in a NDIS service setting
- Explore students’ experiences of the NDIS service placements, how well their learning needs have been met, use of resources and suggestions for change
- ? how to evaluate if they perceived their placement experience to be ‘high quality’?

**Focus Group Schedule**

*Preamble:*

*Thanks for volunteering for this focus group. As you know, your placement was part of a project exploring how student placements fit within the NDIS funding model. The NDIS is causing a lot of changes in the way that service providers design, develop and deliver services. Whether or not it’s possible to host students, and how, is part of those changes.*

*As part of the project, we agreed with the Department of State Development that we’d trial some student placements in services operating under NDIS funding, and evaluate how it went, to be able to identify challenges and organise how to overcome these. It’s important that everyone’s needs are met during this process – clients need a good service, service providers need a good business model, and students need a good learning experience. We’re trying to get around to talk to all of these groups about their perspectives and experiences.*

*There aren’t any right or wrong answers in this group. It’s about what your placement was like, and how you think it could be improved, or areas that you thought ran really well. We plan to use your feedback to benefit students, clients, and service providers in the future. We’ve got a list of topics we’d like to talk about and hear everyone’s thoughts on.*

*Can we start by talking about…*

Tell me a bit about your placement.

**General experience in NDIS placement:**

- You have all been involved in placements that included NDIS funded participants. Were there any notable differences between these placements and others?
- Was there anything about your placement that significantly influenced how you worked with NDIS participants?
- What was most enjoyable about your placement?
- What was most difficult about your placement?
- What did you learn while on this placement?

**Perceptions of student placement quality with NDIS participants/funding:**

- Having quality student placements is important for developing competent therapists, which ultimately support client wellbeing.
- What do you think makes up a quality student placement?
- To what extent do you feel you had a ‘high quality placement experience’? Discuss why you think that was or wasn’t the case. From your (student) perspective, what indicators were there that your placement experience was a quality one?
- Are there any clear indicators of poor quality placements? What might these be?

**Ideas for improvement of student placements with NDIS participants/funding:**

- Was there anything you wished you’d known going into your NDIS student placement?
- If you had a wish list for how your placement could have been improved, what would be on it? Why?
- Did you have any contact with the placement facilitators in your placement? Was this useful or helpful?
- Did you communicate much with other students on other placements in NDIS-settings? Was this helpful to you or not?

**Intention to work in disability and/or with NDIS participants/funding:**

- Considering your experience, do you think you’d like to get a job in the disability sector when you graduate? Why/why not?
- What needed to be different about your placement to change your mind about this?
